# 

**Published:** 1884-05

**Authors:** 


					﻿Vol. V.
/MAY, 1884.
No. 5.
THE
IntUpcndmiprariiüoncr
A MONTHLY JOURNAL
DEVOTED TO
DENTAL AND ORAL SCIENCE.
W. C. BARRETT, M. D„ D. D. S.,
EDITOR.
The New York Dental Journal Association,
PUBLISHERS,
No. 35 West -iötli Street, New Yorlc.
$2.50 Per Annum in Advance, .... Single Copies, 25 Cents.
Entered at the Post-Office, Buffalo, N. Y., as Second-Class matter.
				

